# The New Markers of Early Obesity-Related Organ and Metabolic Abnormalities

**DOI:** 10.3390/ijms232113437

**Published:** 2022-11-03

**Authors:** Agata Ziomber-Lisiak, Kaja Piana, Beata Ostachowicz, Paweł Wróbel, Paula Kasprzyk, Jolanta Kaszuba-Zwoińska, Agnieszka Baranowska-Chowaniec, Kajetan Juszczak, Magdalena Szczerbowska-Boruchowska

**Affiliations:** 1 Chair of Pathophysiology, Faculty of Medicine, Jagiellonian University Medical College, ul. Czysta 18, 31-121 Krakow, Poland; 2 Faculty of Physics and Applied Computer Science, AGH University of Science and Technology, Al. Mickiewicza 30, 30-059 Krakow, Poland; 3 Department of Urology and Andrology, Collegium Medicum, Nicolaus Copernicus University, ul. M. Curie Skłodowskiej 9, 85-094 Bydgoszcz, Poland

**Keywords:** obesity, high-calorie diet, rat, FGF-19, FGF-21, rubidium, TXRF technique

## Abstract

The objective of our study was to identify new markers related to excessive body adiposity and its early consequences. For this purpose we determined serum FGF-19 and FGF-21 concentrations in obese rats, whose role in the pathogenesis of obesity is not yet established. In addition, a total reflection X-ray fluorescence technique was applied to determine the elemental chemistry of certain tissues affected by obesity. Next, the new biochemical and molecular parameters were correlated with well-known obesity-related markers of metabolic abnormalities. Our obese rats were characterized by increased calorie consumption and body adiposity, hypercholesterolemia, elevated levels of liver enzymes and FGF-21, while the level of FGF-19 was reduced. Strong relationships between new hormones and established metabolic parameters were observed. Furthermore, we demonstrated that obesity had the greatest effect on elemental composition in the adipose tissue and liver and that rubidium (Rb) had the highest importance in distinguishing the studied groups of animals. Tissue Rb strongly correlated with both well-known and new markers of obesity. In conclusion, we confirmed serum FGF-19 and FGF-21 as useful new markers of obesity-related metabolic alternations and we robustly propose Rb as a novel indicator of excessive body adiposity and its early consequences. However, further investigations are encouraged to address this clinical issue.

## 1. Introduction

Obesity is a chronic disease as well as a growing health and economic problem all over the world. It is associated with excessive accumulation of adipose tissue, which in turn leads to an increased risk of developing many civilization diseases including cardiovascular (e.g., hypertension, stroke, coronary artery disease) and metabolic (e.g., type 2 diabetes, gout, nonalcoholic fatty liver disease; NAFLD, chronic kidney disease; CKD) disorders as well as cancer [1]. Environmental factors, such as lifestyle favoring a positive energy balance and disturbed balance between energy supply and expenditure are the most prevalent causes of excessive body adiposity. Energy imbalance nowadays is mainly due to widespread access to means of transport and electronic devices as well as to the growing availability of high-calorie food associated with civilization changes [2]. This growing prevalence of overweight/ obesity and its consequent complications prompts the search for new markers of early metabolic alterations typical of excessive body adiposity in order to prevent the occurrence of obesity-related abnormalities and to recognize them at the earliest possible stage of their development. Multiple parameters related to obesity complications are available nowadays, but most of them appear in the advanced stage of the disease when the alterations are profound or irreversible. Except for commonly known metabolic parameters such as fasting glucose, insulin, lipid and lipoprotein concentrations or parameters linked to the liver (aspartate transaminase, AST and alanine transaminase, ALT) or to kidney (microalbuminuria) abnormalities, biological markers of cardiovascular complications have also been proposed such as leptin, ghrelin and adiponectin [3]. In addition, Zang et al. recommended high sensitivity troponin T (hs-cTnT), N-terminal prohormone of brain natriuretic peptide (NT-proBNP), pulse wave velocity (PWV) and central systolic brain pressure (CSBP) for the testing of cardiovascular risk in obese individuals [4].

Recently, the new proteins of the same family members, fibroblast growth factor-19 (FGF-19) and fibroblast growth factor-21 (FGF-21), with pleiotropic effects on development, organogenesis and metabolism have been proposed to be involved in the pathogenesis of obesity [5]. Although some animal studies have suggested the therapeutic potential of FGF-19 and FGF-21 in the treatment of obesity, diabetes or metabolic syndrome [6], still little is known about their role in the metabolic disorders. It was shown that a central administration of the human recombinant FGF-19 can ameliorate insulin resistance and hyperglycemia as well as reduce body weight gain in diet-induced obese mice [6]. The effects of FGF-19 on lipid metabolism, in contrast, differ depending on conditions [6]. FGF-21, expressed in most of the tissues, but predominantly in the liver and in fat, improves insulin sensitivity and therefore glucose tolerance resulting in reduction of blood glucose levels. In addition, FGF-21 administration corrects lipid metabolism leading to decreased blood low-density lipoprotein cholesterol (LDL) and triglyceride levels as well as elevated high density lipoprotein cholesterol (HDL) concentration [7]. Central FGF-21 management is able not only to ameliorate insulin sensitivity, but also to increase energy expenditure resulting in body weight loss in obese rats [7]. On the other hand, Tinkov et al. have shown that not only a biological approach but also molecular measurements may be valuable to detect obesity-related metabolic abnormalities [8]. They revealed that reduced tissue trace elements such as calcium (Ca), iron (Fe), magnesium (Mg), selenium (Se), vanadium (V) and zinc (Zn), as well as increased copper (Cu) levels are associated with obesity. Moreover, a significant relationship between these trace metal abnormalities have coexisted with insulin resistance, atherogenic lipid profile and increased blood pressure. 

Elevated rubidium (Rb) contents were recently found in the hair samples of obese women and these contents were also positively correlated with age and BMI [9]. 

In our study, in order to determine the molecular chemistry of certain tissues and organs known to be affected by excessive body adiposity and by its complications, a total reflection X-ray fluorescence (TXRF) technique was applied [10]. The principle of X-ray fluorescence is based upon the detection of X-rays emitted from sample atoms irradiated with X-rays of higher energy. The energy of the emitted X-rays is indicative of the excited element, thus enabling the identification and quantification of all detectable atoms present in the sample. The advantage of the TXRF method is in its geometry, by which an incident beam of X-rays just grazes the sample, delivering low-background noise. This results in a high-sensitivity measurement of ultra-trace elements i.e., with detection limits in the parts-per-billion range for most elements, that is crucial in the analysis of biological materials. Moreover, the TXRF technique is a multi-elemental analytical tool that enables measurement of all the elements of interest simultaneously. Therefore, the use of the TXRF method can be successfully applied to determine changes of chemical element contents in rat tissues and organs.

The objective of our study was to identify new markers related to excessive body adiposity and its early consequences. For this purpose, in rats with obesity induced by a high-calorie diet (HCD), we determined the serum concentration of the two new hormones—FGF-19 and FGF-21—whose role in the pathogenesis of obesity is suggested, but not yet established. Next, the new biochemical and molecular factors were correlated with well-known obesity-related parameters to find the most appropriate new markers of excessive body adiposity and its metabolic as well as organ abnormalities at the early stage.

## 2. Results

### 2.1. Physiological and Metabolic Parameters

**Figure 1:**
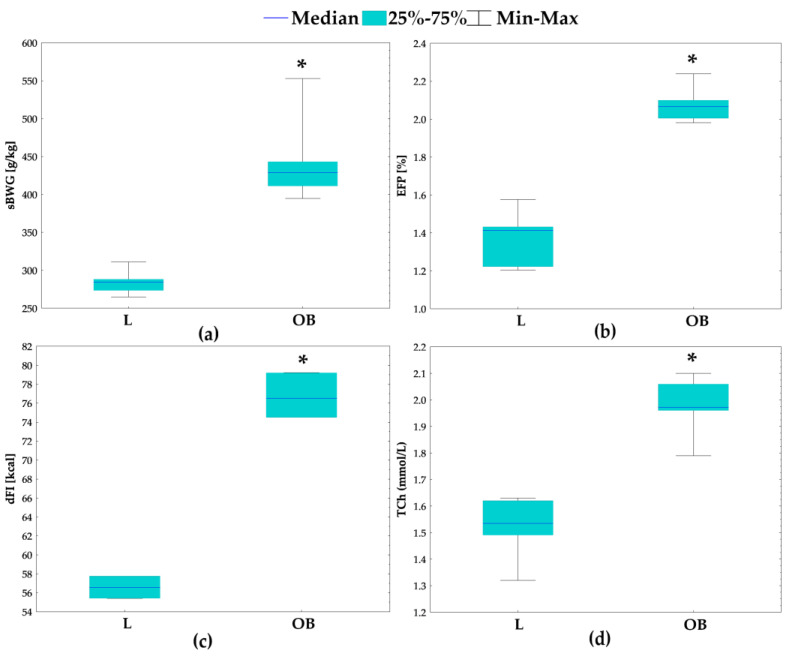

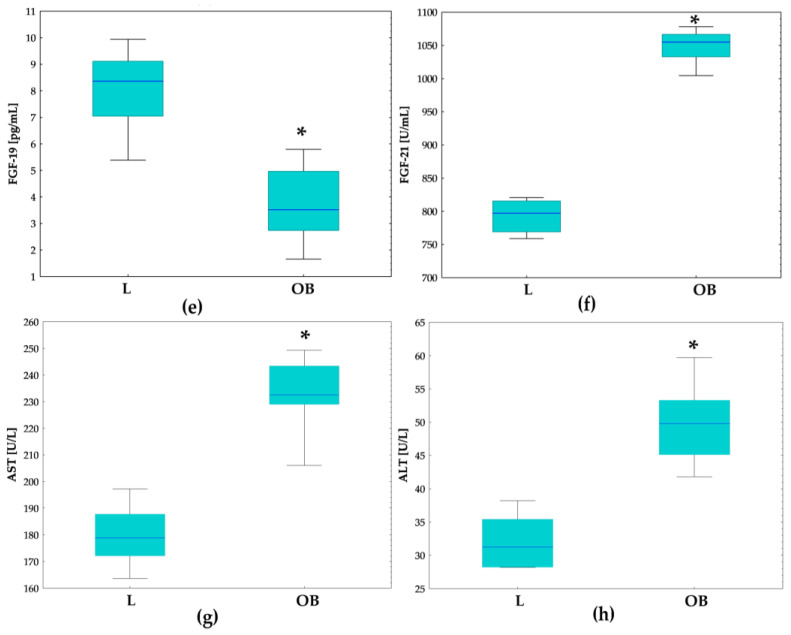
Specific rate of body weight gain (sBWG, g/kg) (**a**), epididymal fat pad (EFP, %) (**b**), daily feed intake (dFI, kcal) (**c**), concentrations of serum: total cholesterol (TCh, mmol/L) (**d**), fibroblast growth factor 19 (FGF-19, pg/mL) (**e**), fibroblast growth factor 21 (FGF-21, U/mL) (**f**), liver aspartate transaminase (AST, U/L) (**g**) and liver alanine transaminase (ALT, U/L) (**h**). L—lean intact (*n* = 6); OB—obese intact (*n* = 6); * *p* < 0.05 vs. L.

**Figure 2:**
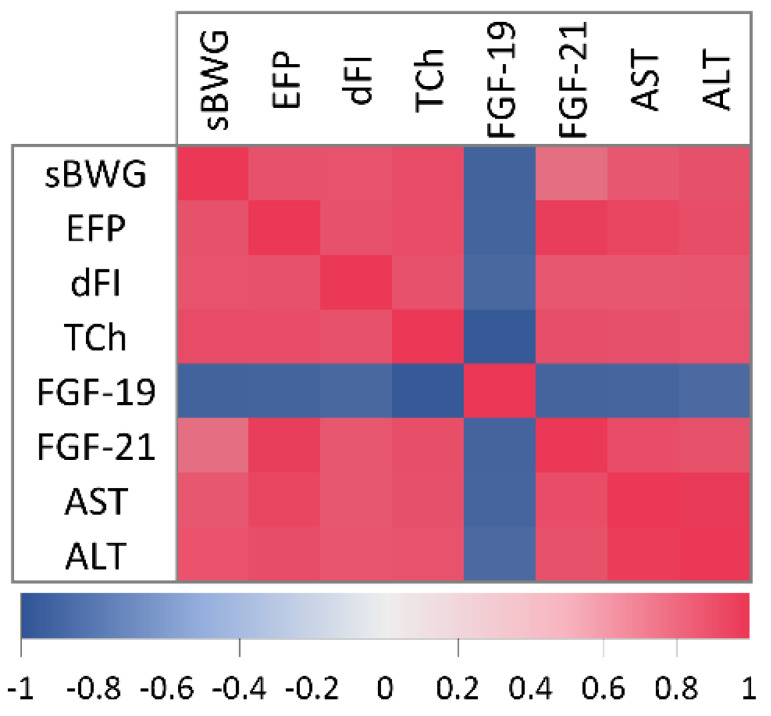
Pearson’s or Spearman’s correlation coefficients between physiological and metabolic parameters. sBWG—specific rate of body weight gain, EFP—epididymal fat pads, dFI—daily feed intake, TCh—total cholesterol, FGF-19—fibroblast growth factor 19, FGF-21- fibroblast growth factor 21, AST—liver aspartate transaminase, ALT—liver alanine transaminase, All correlations are statistically significant (at the *p*-levels < 0.05).

As we expected, the rats on a HCD gained their body weight faster than the lean (L) animals fed a standard chow as the obese (OB) animals had a higher specific rate of body weight gain (sBWG, g/kg; *p* = 0.002) during the study (Figure 1a). In spite of an insignificant difference in the end body weight (eBW, g) between the L and OB groups, based on the Mann Whitney U test (436 g (382 ÷ 468) vs. 476 g [429 ÷ 536], respectively), body adiposity, measured as the epididymal fat pad percentage of eBW (EFP, %) at the end of the study, was higher in the OB than in the L group (*p* = 0.002) (Figure 1b). Moreover, we observed significantly higher daily feed intake (dFI, kcal) for the rats on a HCD than for their lean counterparts (Figure 1c). Increased body weight gain went along with higher daily feed intake (r_S_ = 0.84, *p* < 0.05) (Figure 2) indicating overconsumption as being a dominant cause of obesity observed in our rats. Based on the Mann Whitney U test, we also detected different ranges of serum total cholesterol (TCh; *p* = 0.002), AST (*p* = 0.002) and ALT (*p* = 0.002) in our rats depending on the diet consumed—OB had significantly higher ranges of all three measured parameters when compared with L (Figure 1d,g,h). Interestingly, serum FGF-19 levels were lower (*p* = 0.002), while FGF-21 levels were higher (*p* = 0.002) in the rats on a HCD than in their lean counterparts (Figure 1e,f, respectively).

### 2.2. Correlation Analysis of Physiological and Metabolic Parameters

Based on correlation analysis, we observed high correlations (r ≥ 0.70) between measured physiological and metabolic parameters for most cases (c.f. Figure 2). sBWG highly correlated with both EFP and dFI (r_S_ = 0.85, *p* < 0.05 and r_S_ = 0.84, *p* < 0.05, respectively) and strong relationships between sBWG and TCh, AST or ALT (r_S_ ≥ 0.81, *p* < 0.05) were also noted. In addition, sBWG highly correlated with FGF-19 (r_S_ = −0.89, *p* < 0.05) and moderately with FGF-21 (r_S_ = 0.66, *p* < 0.05). Strong relationships between EFP (r_P_/s ≥ 0.85, *p* < 0.05) or dFI (r_P_/s ≥ 0.81, *p* < 0.05) and other physiological and metabolic parameters were also detected. Interestingly, TCh highly correlated with sBWG (r_S_ = 0.89, *p* < 0.05), dFI (r_S_ = 0.85, *p* < 0.05), EFP (r_P_ = 0.88, *p* < 0.05), AST (r_P_ = 0.86, *p* < 0.05), ALT (r_P_ = 0.84, *p* < 0.05) and even FGF-21 (r_P_ = 0.86, *p* < 0.05), but the strongest inverse relationship was found to be between serum TCh and FGF-19 (r_P_ = −0.96, *p* < 0.05). In contrast, serum FGF-21 positively related most to EFP (r_P_ = 0.97, *p* < 0.05). In addition, both FGF-19 and FGF-21 highly correlated with AST (r_P_ = −0.87, *p* < 0.05 vs. r_P_ = 0.88, *p* < 0.05, respectively) and ALT (r_P_ = −0.84, *p* < 0.05 vs. r_S_ = 0.84, *p* < 0.05, respectively). It was not a surprise to find a very dependable relationship between AST and ALT (r_P_ = 0.98, *p* < 0.05). It should be emphasized that serum FGF-19 was the only parameter negatively correlated with the other tested markers.

### 2.3. Tissue Elemental Concentrations

**Figure 3:**
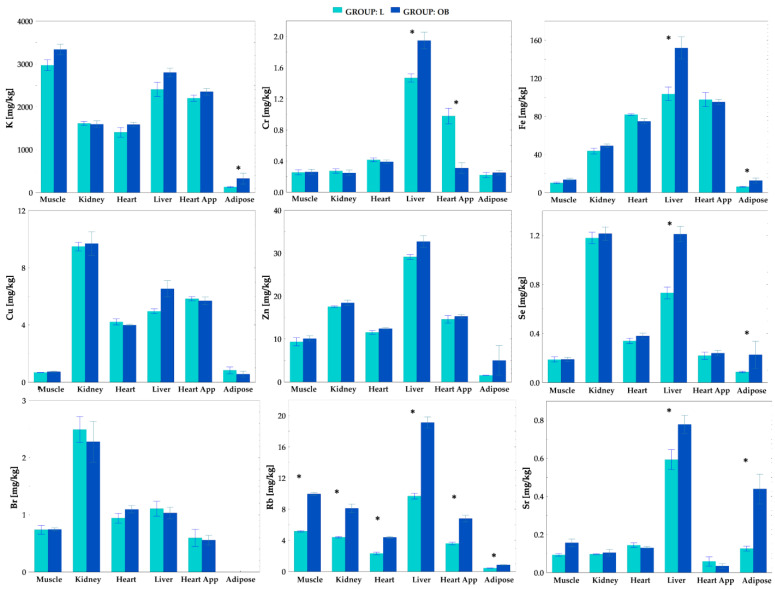
The comparison of average elemental concentrations (mg/kg) in rat organs and tissues between lean intact (L) and obese (OB) individuals. *—statistically significant differences (at *p*-levels < 0.05).

Based on the TXRF analysis we determined the mean concentrations of all the detectable elements, i.e., K, Cr, Fe, Cu, Zn, Se, Br, Rb and Sr, in the organs’ tissues for the L and OB individuals. The results of the comparative analysis for the studied groups of animals are presented in Figure 3. We observed that obesity induced by a HCD had the greatest effect on elemental composition in the adipose tissue and liver. In particular, in the case of the adipose tissue levels of K (*p* = 0.008), Fe (*p* = 0.02), Se (*p* = 0.04), Rb (*p* = 0.005) and Sr (*p* = 0.03), they were found to be significantly higher in the OB group as compared to the L group. Moreover, a significant increase in Cr (*p* = 0.005), Fe (*p* = 0.01), Se (*p* = 0.005), Rb (*p* = 0.005) and Sr (*p* = 0.03) concentrations was found in the livers of the obese animals. In the muscle tissue both Rb (*p* = 0.005) and Sr (*p* = 0.04) were significantly higher in OB animals. However, in the apical region of the heart, a decreased Cr level (*p* = 0.008) and an increased Rb level (*p* = 0.005) were found due to obesity. For the other tissues examined, i.e., the heart (*p* = 0.005) and the kidney (*p* = 0.005), only the level of Rb was found to be significantly elevated in the obese animals. It should be emphasized that in all examined organs the level of Rb was significantly elevated. This is the only element for which such an observation was found.

### 2.4. Intake of Elements with Food Consumption

**Figure 4:**
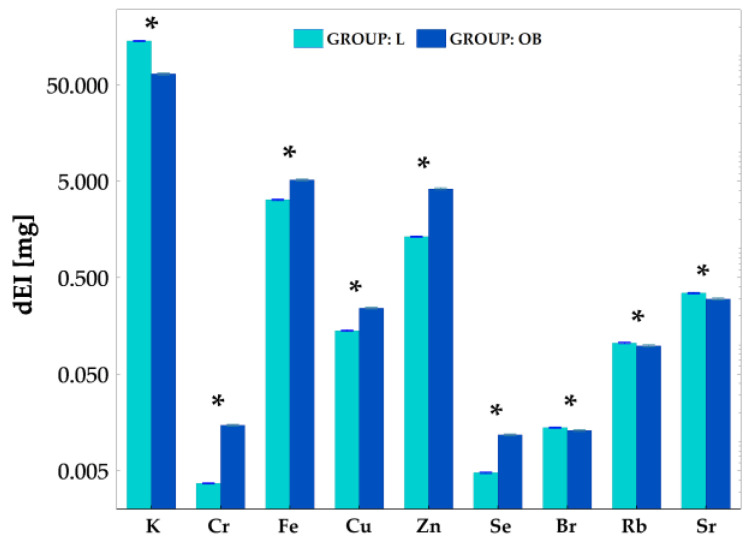
The comparison of the average daily intake of elements with food (dEI) (mg) between lean intact (L) and obese (OB) individuals. *—statistically significant correlation (at the *p*-levels < 0.05).

To check the direct effect of the food consumed by the animals in both groups, the feed was also subjected to elemental analysis. Then, based on the determined concentrations of elements in the feed as well as the daily food intake by individuals, the average daily intake of elements (dEI, mg) for both groups was calculated (c.f. Figure 4). Based on the Mann Whitney U test, there were significant differences in the levels of daily element intake between the study groups for all elements. However, the dEI for K (*p* = 0.005), Br (*p* = 0.02), Rb (*p* = 0.02) and Sr (*p* = 0.005) was lower in the OB group than in the L group. For the other elements, i.e., Cr (*p* = 0.005), Fe (*p* = 0.005), Cu (*p* = 0.005), Zn (*p* = 0.005) and Se (*p* = 0.005), an opposite relationship was observed.

It should be noted that the increased intake of Cr and Se with food was accompanied by elevated levels of these elements in the liver. Similarly, increased Se and Fe intake from food in the OB group went hand in hand with higher levels of these chemical elements in adipose tissue. In contrast, a decrease in Cr level was observed in the apical region of the heart despite the increased daily intake of this element with food.

Interestingly, despite a significantly lower dEI of Rb in the OB group, higher levels of this element were found for obese animals in all their organs which were examined compared to those organs of the lean intact individuals. A similar relationship between dEI and organ element content was observed for Sr, but only in the case of liver and adipose tissue.

### 2.5. Multivariate Discriminant Analysis—Complex Elemental Differentation of Lean and Obese Animals

Apart from the intergroup comparison of elemental contents, we also used complex information provided by the TXRF technique applying multiple discriminant analysis (MDA). As presented in Table 1, the MDA confirmed the greatest importance of Rb in distinguishing the studied groups of animals. For all organs and tissues, this chemical element showed the greatest significance in discriminating between obese and lean animals (the lowest values of partial Wilks’ lambda—PWL). In addition, among the elements that were significant in differentiating between the groups, but were found to be of lesser importance, the following were identified: for the liver—Zn, for the heart apex—Cr, for the skeletal muscle—Br, for the kidney—Sr and Zn, for the rest of the heart—K. In the case of the adipose tissue, Rb was the only element statistically significant in the discrimination.

### 2.6. Intragroup Elemental Relationships

**Figure 5:**
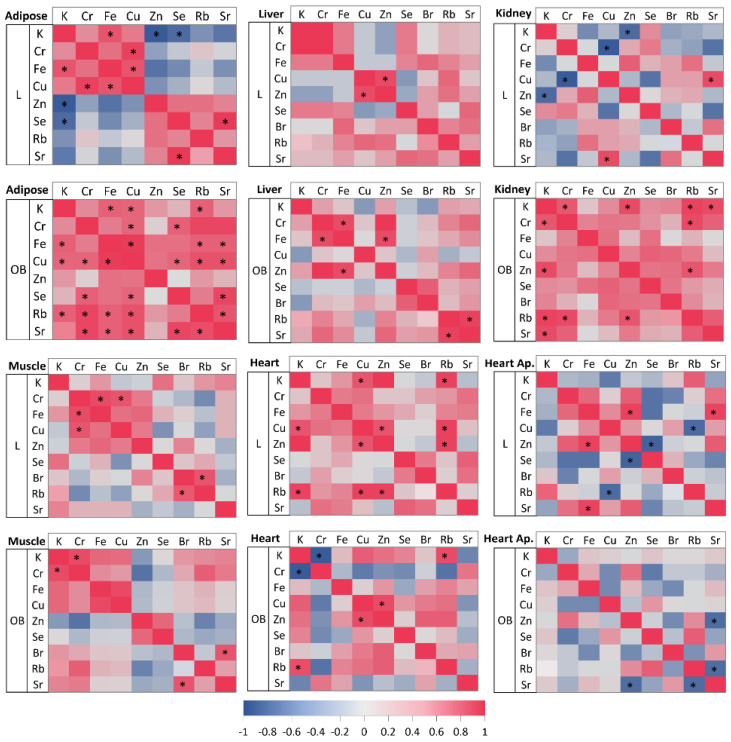
Pearson’s or Spearman’s correlation coefficients for elemental relationships in organs and tissues for lean intact (L) and obese (OB) groups. *—statistically significant correlation (at the *p*-levels < 0.05).

To identify potential sources of the observations obtained, we examined inter-elemental relationships in organs occurring in the animals fed standard or high-calorie diets. In the liver tissue, strong correlations were found between Cu and Zn (r_S_ = 0.86, *p* < 0.05) in the lean intact animals (Figure 5), while, in the case of obese individuals, Cr and Fe (r_S_ = 0.85, *p* < 0.05), Zn and Fe (r_S_ = 0.86, *p* < 0.05), as well as Rb and Sr (r_S_ = 0.84, *p* < 0.05) were strongly correlated (Figure 5). In addition, the study indicated (c.f. Figure 5) that in the heart apex of lean animals a very strong positive correlation between Fe and Zn (r_S_ = 0.94, *p* < 0.05) or between Fe and Sr (r_S_ = 0.92, *p* < 0.05) exists. Furthermore, a strong negative correlation was observed between Rb and Cu (r_S_ = −0.83, *p* < 0.05) and between Zn and Se (r_S_ = −0.86, *p* < 0.05). In contrast, a strong negative correlation between the levels of Rb and Sr (r_S_ = −0.81, *p* < 0.05) and between Zn and Sr (r_S_ = −0.81, *p* < 0.05) in the heart apex of the obese individuals was observed. A high positive association between Fe and Cr (r_S_ = 0.86, *p* < 0.05), between Cu and Cr (r_S_ = 0.83, *p* < 0.05) as well as between Rb and Br (r_S_ = 0.83, *p* < 0.05) was also found in the skeletal muscle of the L group (Figure 5). In contrast, in the skeletal muscle of obese animals, the levels of Cr and K (r_S_ = 0.86, *p* < 0.05) as well as Br and Sr (r_S_ = 0.83, *p* < 0.05), showed very strong positive correlations (Figure 5). In the kidney of lean animals (c.f. Figure 5), strong negative correlations between K and Zn (r_S_ = −0.83, *p* < 0.05), as well as between Cu and Cr (r_S_ = −0.94, *p* < 0.05), were observed. Meanwhile, Cu and Sr were strongly positively correlated (r_S_ = 0.86, *p* < 0.05). It was further shown (Figure 5) that, in the kidney of obese individuals, the level of K strongly positively correlated with Cr (r_S_ = 0.81, *p* < 0.05), Zn (r_S_ = 0.86, *p* < 0.05), Rb (r_S_ = 0.94, *p* < 0.05) and Sr (r_S_ = 0.86, *p* < 0.05). In addition, the level of Cr strongly positively correlated with the levels of Rb (r_S_ = 0.90, *p* < 0.05) and additionally Rb with Zn (r_S_ = 0.83, *p* < 0.05). In the rest of the heart of group L (c.f. Figure 5), a very strong correlation between K and Cu (r_S_ = 0.83, *p* < 0.05), K and Rb (r_S_ = 0.94, *p* < 0.05), Zn and Rb (r_S_ = 0.94, *p* < 0.05), Rb and Cu (r_S_ = 0.94, *p* < 0.05) as well as Cu and Zn (r_S_ = 0.83, *p* < 0.05) was shown. In the OB group, K was similarly found to be strongly positively correlated with Rb (r_S_ = 0.86, *p* < 0.05), as was the case also in the L group. Moreover, in the same individuals, the level of Cr showed a negative strong correlation with K (r_S_ = −0.94, *p* < 0.05). In addition, in this case, Cu similarly to Rb was found to correlate strongly with the level of Zn (r_S_ = 0.86, *p* < 0.05). In the adipose tissue of obese animals, as shown in Figure 5, strong positive correlations were found between Fe levels and K (r_S_ = 0.83, *p* < 0.05), Cu (r_S_ = 0.99, *p* < 0.05), Rb (r_S_ = 0.83, *p* < 0.05) and Sr (r_S_ = 0.83, *p* < 0.05) levels. Furthermore, Rb strongly related to K (r_S_ = 0.83, *p* < 0.05), Cr (r_S_ = 0.94, *p* < 0.05), Cu (r_S_ = 0.90, *p* < 0.05) and Sr (r_S_ = 0.86, *p* < 0.05), while Cu correlated additionally with Cr (r_S_ = 0.81, *p* < 0.05), Se (r_S_ = 0.81, *p* < 0.05) and Sr (r_S_ = 0.90, *p* < 0.05). In addition, Se and Sr also strongly positively correlated (r_S_ = 0.94, *p* < 0.05). In contrast, in the adipose tissue of lean animals, K strongly negatively correlated with Zn (r_S_ = −0.94, *p* < 0.05), Se (r_S_ = −0.83, *p* < 0.05) and Fe (r_S_ = 0.83, *p* < 0.05) (positive correlation). In addition, Cr and Cu (r_S_ = 0.94, *p* < 0.05), Se and Sr (r_S_ = 0.96, *p* < 0.05) and Cu and Fe (r_S_ = 0.82, *p* < 0.05) were similarly strongly positively correlated (cf. Figure 5).

### 2.7. Correlation of Rb with Physiological and Metabolic Parameters

**Figure 6:**
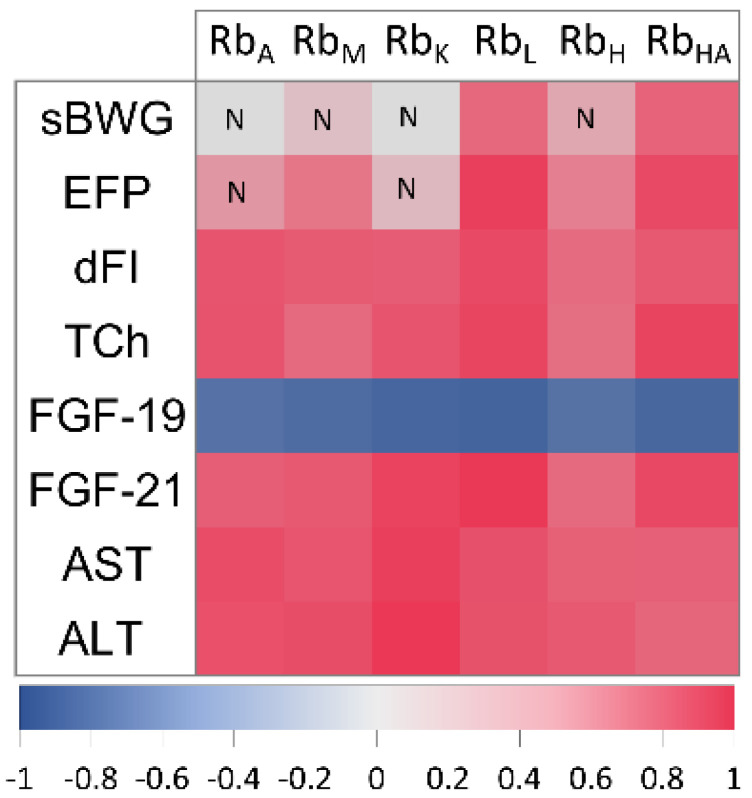
Pearson’s or Spearman’s correlation coefficients for relationships between rubidium (Rb) concentrations in rat organs/tissues and physiological or metabolic parameters. sBWG—specific rate of body weight gain, EFP—epididymal fat pads, dFI—daily feed intake, TCh—total cholesterol, FGF-19—fibroblast growth factor 19, FGF-21—fibroblast growth factor 21, AST—liver aspartate transaminase, ALT—liver alanine transaminase, A—adipose tissue, M—muscle, K—kidney, L—liver, H—heart, HA—heart apex, N—statistically insignificant correlation. Correlations other than marked “N” are statistically significant (at the *p*-levels < 0.05).

Additionally, taking into account the obtained observations for Rb, correlations of this element with some physiological and metabolic parameters were determined (c.f. Figure 6). Rb in the adipose tissue significantly highly correlated (r_P/S_ ≥ ±0.78, *p* < 0.05) with all the measured metabolic (TCh, AST, ALT, FGF-19, FGF-21) and physiological (dFI) parameters, except sBWG and EFP. Rb in the skeletal muscle also correlated with the above parameters and, in addition, a significant relationship between Rb and EFP was noted (r_p_ = 0.70, *p* < 0.05). Rb in the liver, apart from all the mentioned blood examinations, correlated with sBWG (r_s_ = 0.76, *p* < 0.05), EFP (r_P_ = 0.92, *p* < 0.05) and dFI (r_S_ = 0.88, *p* < 0.05). Among the metabolic parameters, FGF − 21 correlated with the liver Rb the most (r_p_ = 0.95, *p* < 0.05). Similar relationships were observed in the case of Rb contents in the kidney, heart and heart apex tissues. Interestingly, heart apex Rb strongly correlated with the all blood metabolic and even physiological parameters such as sBWG (r_S_ = 0.78, *p* < 0.05), EFP (r_P_ = 0.88, *p* < 0.05) and dFI (r_S_ = 0.82, *p* < 0.05). In contrast, the rest of the heart tissue Rb content did not relate to sBWG, but rather to EFP (r_P_ = 0.66, *p* < 0.05), dFI (r_S_ = 0.74, *p* < 0.05) and other blood obesity-related markers (r_P_ ≥ ±0.73, *p* < 0.05). Kidney Rb highly correlated with all the tested metabolic parameters (r_P_ ≥ ±0.81, *p* < 0.05) and dFI (r_S_ = 0.80, *p* < 0.05), except sBWG and EFP. In all cases, tissue Rb negatively correlated with FGF-19. Given blood metabolic parameters, the following strongest correlations with tissue Rb were found: AST and ALT in the adipose tissue (for both r_P_ = 0.87, *p* < 0.05), skeletal muscle (r_P_ = 0.83, *p* < 0.05 and r_P_ = 0.86, *p* < 0.05, respectively), kidney (r_P_ = 0.92, *p* < 0.05 and r_P_ = 0.95, *p* < 0.05, respectively) and heart (r_P_ = 0.79, *p* < 0.05 and r_P_ = 0.82, *p* < 0.05, respectively), FGF21 in the liver (r_P_ = 0.95, *p* < 0.05) and kidney (r_P_ = 0.91, *p* < 0.05) and TCh (r_P_ = 0.90, *p* < 0.05) and FGF-21 (r_P_ = 0.89, *p* < 0.05) in the heart apex.

## 3. Discussion

Obesity, a disease with a rapidly increasing frequency worldwide, is not only the problem of excessive body adiposity, but it also has health consequences such as heart disease, stroke, diabetes, high blood pressure, liver disease, asthma, sleep apnea, gallstones, kidney stones, infertility, etc. [11]. Because the treatment of obesity and its chronic complications is long-lasting, expensive and not-effective in many cases, identification of early markers of adult body adiposity is useful for clinicians to prevent severe and irreversible health consequences and for research to investigate the origin of obesity. Unhealthy cholesterol (hypercholesterolemia or dyslipidemia), for example, commonly observed in obese individuals, is an established early marker of premature coronary heart diseases, along with hypertension, smoking, genetic predisposition and others risk factors [12]. Increased blood glucose levels and insulin resistance frequently diagnosed in patients with obesity predict the future risk of type 2 diabetes [13] while microalbuminuria, present not only in the individuals with obesity, diabetes and/or hypertension, but also in healthy subjects, is an independent risk factor for cardio-vascular disease [14]. Increased ALT and AST in obese individuals is usually a marker of NAFLD, an early stage of the liver disorder which can progress to nonalcoholic steatohepatitis (NASH) or even liver cirrhosis [15]. Due to multiple and complex mechanisms involved in the pathogenesis of obesity (e.g., a brain-gut axis, vagus reflex), more and more factors are proposed to be useful in the explanation of obesity and the development of its consequences.

Our rats on a HCD had a higher body weight gain and body adiposity than their lean counterparts and that went along with a higher feed intake indicating overconsumption as a dominant cause of obesity. Male rats were used in the study because, on the basis of our own [16,17,18,19] and other experiences [20], they were shown to gain weight faster on a HCD compared to female rats. Moreover, most of the data on the elemental composition of tissues cited in our study concerned male specimens. Taking into account the limited number of records on the issue raised at work, it seems that this was a justified choice. Increased body fat was accompanied by significantly higher serum TCh, AST and ALT levels confirming the causative relationship between obesity and metabolic abnormalities. Our previous research found TCh to be the best indicator of obesity-related metabolic alternations in both male and female obese Wistar rats [18,21]. Among the determined parameters of lipid disorders, TCh differed significantly depending on the body fat and its level was highly correlated with the LDL concentration. HDL and triglycerides did not differ with body weight. AST and ALT are markers of liver dysfunction, especially NAFLD, that are quite common in obesity [15] and our study confirmed the presence of that relationship at the early stage of obesity development. In contrast, the rats with obesity induced according to our protocol (the chow with increased fat contents), showed no impaired glucose metabolism (no published data), which could be a matter of obesity induction time or/ and diet composition. The fact that there was no significant difference in the end body weight between the tested groups observed in our study indicates that total body weight is not a precise parameter reflecting body adiposity or energy status. dFI highly correlated not only with established markers of metabolic abnormalities such as TCh, AST and ALT, but also with the new hormones such as FGF-19 and FGF-21. Interestingly, serum FGF-19 levels were reduced, while FGF-21 levels were elevated in our obese animals, comparing them with our lean subjects, a result which is in line with the previous reports [22,23]. It is suggested that although FGF-19 and FGF-21 are activated under different conditions, they show a similar function in their controlling of glucose and insulin metabolism, energy and weight balance as well as lipid concentrations, particularly in metabolic disorders such as obesity or diabetes [22,23]. Both clinical and animal studies have demonstrated an inverted relationship between blood FGF-19 and obesity [22]. Serum FGF-19 levels were lower in obese adolescence with NAFLD compared with healthy controls and negatively related to the probability of NASH [24]. Decreased body weight and blood glucose levels after the administration of the human recombinant FGF-19 was observed in HFD-induced obese mice [23]. In addition, FGF-19 transgenic mice were resistant to HFD-induced obesity and increased fat accumulation [25]. In contrast, obese human and rodent subjects have increased, rather than reduced, blood FGF-21 levels [22,26]. This is probably due to resistance to FGF-21 action and could be explained by the down-regulation of FGFR1 and β-Klotho demonstrated when shown in HFD-induced obese mice [27]. An FGF-21-resistance state can be reversed by weight loss or hypoglycemic therapy [28] and FGF-21 transgenic mice or administration of exogenous FGF-21 resulted in resistance to diet-induced obesity, pointing at FGF-21 as a potential anti-obesity molecule [27].

In our study, both FGF-19 and FGF-21 highly correlated (r_P/S_ ≥ ±0.81) with most of the tested physiological and metabolic parameters and the highest association between FGF-19 and TCh (r_P_ = −0.96) and between FGF-21 and EFP (r_P_ = 0.97) was detected. The only difference was in the case of sBWG which highly inversely correlated with FGF-19 (r_S_ = −0.89), but only moderately positively with FGF-21 (r_S_ = 0.66). These findings suggest that the new hormones are comparably effective in detecting excessive body adiposity and obesity-related metabolic abnormalities as well-known and established markers. Both of these hormones also highly negatively correlated with each other (r_P_ = −0.88), which confirms their functional similarity despite their activation in different conditions. FGF-19, secreted in the gut during feeding, plays an important role in regulating lipid and glucose metabolism. Decreased serum FGF-19 levels during fasting was shown to be associated with the development of NAFLD in obese adolescents [24]. Treating obese mice with FGF-19 on the one hand decreased the transcription of a series of lipogenesis-associated genes [29], but on the other hand increased serum triglicerydes (TGs) and TCh levels [30]. The dual functions of FGF-19, both lipid-raising and -lowering effects, could be attributed to different binding receptors and target tissues [30]. It was shown that FGF-19 transgenic mice were resistant to glucose intolerance and hyperinsulinemia induced by a high-fat diet (HFD) [25]. Similarly, the FGF-19 treatment reversed the development of impaired glucose metabolism in either HFD-fed, ob/ob or FGF15-KO mice [23,31,32]. Unlike FGF-19, FGF-21 is mainly secreted at the late stage of fasting [33]. Clinical studies have shown that serum FGF-21 levels positively correlated with obesity and fatty liver [34,35] and that lipid infusion increased FGF-21 levels [36]. Therefore one may suggest that increased FGF-21 levels might be an adaptive protective response to lipo-toxicity. Similarly to FGF-19, FGF-21 demonstrates the opposite effects on lipid metabolism, which could be explained by diverse regulatory roles of FGF-21 in various nutritional states [37]. FGF-21 transgenic mice had significantly decreased levels of serum and hepatic TGs compared with WT mice [38] but on the other hand, FGF-21 induced adipocyte differentiation and lipogenesis in obese rodents [39]. Therefore, it is proposed that in the fasted state FGF-21 leads to hyperglycemia by stimulating lipolysis, ketogenesis, gluconeogenesis and increasing insulin sensitivity [40], while in the fed state, FGF-21 induces hypoglycemic effects by stimulating lipogenesis and adipocyte differentiation [39].

There is some evidence that metals and trace elements may impact body weight. For example, arsenic and cadmium have been associated with changes in adipose tissue physiology and glucose metabolism [41,42]. The elements lead, cadmium, arsenic and mercury have been related to increased oxidative stress [42,43], while cadmium, mercury, arsenic, lead, manganese and zinc are suggested to act as endocrine disruptors [44]. Essential metals (e.g., cobalt, copper, chromium, iron, manganese, molybdenum, nickel, selenium and zinc) play a known physiological role in the body and are necessary at certain levels in order to avoid deficiency-related health consequences [45,46]. However, the changes in the elemental composition of biological tissues can result from certain pathological conditions [47,48].

In our study, a HCD had the greatest effect on elemental composition in the adipose tissue and in the liver—the epididymal fat K, Fe, Se, Rb and Sr and liver Cr, Fe, Rb and Sr were significantly higher in the OB group as compared to the L group. In the reminder of the examined samples (heart and heart apex, skeletal muscle and kidney) the Rb level was found to be significantly elevated due to obesity, while decreased Cr was found in the apical region of the heart and increased Sr in the skeletal muscle. It should be highlighted that Rb was the only measured element significantly elevated in all of the tested tissues. Because one could imply that the differences in tissue elemental composition come from the various diets consumed, the feed was also subjected to elemental analysis and the average daily intake (dEI) of elements for the both groups was calculated. Indeed there were significant differences in the levels of daily element intake between the study groups. Cr, Fe, Cu, Zn and Se intake was higher in the OB group and it was accompanied by the elevated liver Cr and Se as well as fat Fe concentrations. These findings may suggest that increased tissue element concentration in the obese animals resulted from their higher consumption with chow and therefore accumulation in the liver and the adipose tissue, which are known as the storage organs. In contrast, the dEI for K, Br, Rb and Sr was even lower in the OB group, than in the L group despite increased (K, Rb and Sr) or unchanged (Br) tissue concentration, which suggests organ elemental redistribution due to obesity-related alternations. Sr, for example, accumulated in the liver, muscle and adipose tissue, while K did so in the fat. Interestingly, despite significantly lower dEI of Rb in the OB group, higher levels of this element were found for obese animals in all examined organs compared to those levels for the lean intact individuals. It should be stressed that Rb was the only examined element elevated in all of the tested tissues of the obese animals despite its decreased dietary consumption, therefore we robustly propose Rb as a new molecular marker of obesity-related alternations. Our hypothesis is additionally supported by the finding that strong relationships between Rb and other established markers of obesity-related metabolic disturbances were shown in our study. We demonstrated that tissue Rb highly correlated with most of the physiological and biochemical parameters (c.f. Figure 6). Interestingly, tissue Rb, regardless of the type of organ tested, highly related to dFI indicating the diet consumed to be a main source of this element in the body. In contrast, only Rb in the liver and heart apex strongly correlated with both sBWG and EFP, suggesting that Rb content in these tissues might reflect body adiposity. Rb in all the measured tissues highly related to AST and ALT, indicating that obesity-related liver abnormalities are associated with nonspecific tissue Rb increase. TCh highly correlated with Rb content in all the measured samples, but the strongest relations (r_P_ = 0.90) were found between TCh and Rb in the liver and heart apex. This finding once again points at the liver and the heart apex as being the tissue the most affected by obesity-related body Rb redistribution. Indeed, a similar observation was made given serum FGF-19 and FGF-21. Both new obesity-related hormones correlated with Rb in the liver and the heart apex most. In all cases the tissue Rb, similarly to other measured obesity-related markers, inversely correlated with FGF-19.

The biological role of Rb remains understudied. Because Rb resembles K, it may have a role similar to K. The two elements are found together in minerals and soils, although K is much more abundant than Rb. A normal human adult body contains about 300–500 mg of Rb in all its tissues, which is more than other ultra-trace elements. Rb is present in almost all biological systems due to its ability to exchange with K, mainly in the body’s intracellular fluid, and therefore the metabolism of the two ions are closely related [49]. A neurophysiological function of Rb is also proposed. The Rb content differs in particular regions of the brain and Rb has been reported to have enhanced the turnover of brain norepinephrine and cause electroencephalogram activation in rats and monkeys [50,51]. Rb has also been shown to exert several biological and pharmacological effects similar to those of many of the classic antidepressant drugs [52,53]. Rb and K levels were found to be significantly decreased in the brains of people affected by Alzheimer’s disease [54]. Elevated Rb contents were recently found in the hair samples of obese women and this element was also found to be positively correlated with age and BMI [9]. However, these are the only few available pieces of data describing the association of tissue Rb content with obesity and our findings are in accordance with these reported findings. Rb concentration was shown to be elevated in all the measured tissues due to obesity in our study. Moreover, the liver and heart apex Rb content positively correlated with both body weight gain and body adiposity. Because Rb intake was lower in the obese than in the lean animals, our pioneering results cannot be simply explained by increased Rb consumption with a HCD, but rather by redistribution of this element within the body. Consequently, further examinations with Rb content assessment in other tissues e.g., brain or bones are encouraged, which would probably give us additional information about the Rb role in the pathogenesis of obesity. In addition, with such scarce literature data of the discussed scientific field, it is untimely to speculate if the body Rb redistribution is the consequence or rather the cause of excessive body adiposity, especially considering the reported neurophysiological action of this element.

## 4. Materials and Methods

### 4.1. Study Design

Twelve male 18-week-old albinos Wistar rats with initial body weight of 332 ± 25 g were used in the study. The animals were housed in plastic cages (two rats per cage), in an air-conditioned room with a 20–24 °C temperature, 55–65% humidity and a 12:12-h dark/light cycle, with ad libitum feeding and drinking water throughout the study (6 weeks). The rats were randomized into two numerically equal groups. The groups differing in their specific diet consumed: one group was fed on standard chow pellets (*n* = 6) (carbohydrates with ash and minerals 67%, proteins 25% and fats 8%; energy 2.75 kcal/g; Labofeed B, Kcynia, Poland) while the other group was given a high-calorie diet (HCD) (*n* = 6) (carbohydrates with ash and minerals 46%, proteins 32%, fats 22%; energy 4.7 kcal/g; Perform, Opti Life, Kronen, Belgium). It should be underlined that the HCD contained nearly three-fold more fats than the standard chow [44]. The experiments were conducted in accordance with the National Guide for the Care and Use of Laboratory Animals and were approved by the Local Ethical Committee on Animal Testing at the Jagiellonian University in Krakow, Poland (approval no. 157/2013). According to the 3R principles, the number of rats used for the experiment was reduced to the necessary minimum.

### 4.2. Body Weight and Feed Intake Measurements

Dietary intake and body weight were recorded twice a week throughout the course of the study using a digital scale. The rats, according to the specific diet consumed, were randomly assigned to the lean group (L) with an initial body weight (BW0) of 339 g (300 ÷ 370) or to the obese group (OB) with a BW0 of 325 g (302 ÷ 373). Both specific rate of body weight gain (sBWG; g/kg) and daily feed intake (dFI; kcal) were calculated according to the protocol described in our previous study [21].

At the end of the experiment, the animals were sacrificed. Immediately after decapitation, the tissues of interests were rapidly excised, put into chemically inert vials and transferred to a freezer operating at −80 °C. The specimens were stored under these conditions until the time came for biochemical and spectroscopic analysis. Our research was carried out on the blood and certain tissues that are usually affected in obesity: the intraabdominal white adipose tissue (epididymal fat pads, EFPs), the liver, skeletal muscle, kidney, heart apex and the rest of the heart.

### 4.3. Biochemical Analysis of Some Blood Metabolic Parameters

Serum levels of fibroblast growth factor 19 (FGF-19, pg/mL) and fibroblast growth factor 21 (FGF-21; U/mL) were measured using conventional rat ELISA kits (Cloud-Clone Corp., Huston, USA and BioVendor, Czech Republic, respectively). Serum total cholesterol (TCh) (mmol/L) was assessed with the enzymatic method, while aspartate transaminase (AST) and alanine transaminase (ALT) was determined with the kinetic method with NADH and TRIS buffer, according to IFCC, without pyridoxal phosphate, on a Roche Cobas c501 analyzer (Roche Diagnostics GmbH, Mannheim, Germany).

### 4.4. Tissue Sample Preparation for the TXRF Analysis

Prior to the TXRF analysis the tissues were defrosted and then 100 ÷ 200 mg samples were placed in acid digestion bombs (Anton Paar). To each sample nitric acid (V) supra-pure quality (0.5–0.6 mL) and per-hydrol (50 µL) were added. The bombs were carefully sealed and placed in an oven for 8 h at 200 °C. For quantitative elemental analysis, after completing the digestion, Ga solution (1000 mg/L, Merck) was added as an internal standard. To each sample, a 10 µL Ga solution was added to achieve the final concentration of about 40–95 mg/L depending on the mass of the sample. Then 6 µL of the solution was dropped on the reflector and carefully dried on the hot plate (60 °C) in the clean bench. For each sample, three replicates were prepared.

Elemental concentrations were measured using a Total Reflection X-ray Spectrometer, Nanohunter II (Rigaku, Japan). The measurements were performed with the use of an Mo X-ray tube, operating at 55 kV voltage and a current of 12 mA. A live time was equal to 1000 s.

Additionally, the preparation and measurement procedures used for the tissues were also applied to the animal feed (standard and high calorie, 250 mg each).

### 4.5. Data Treatment and Statistical Analysis

For each tissue/animal feed sample, mean values of elemental concentrations from three replicates were calculated with Microsoft Excel. Then, the element concentration values determined in this way were used in further statistical analyses.

In cases of intergroup comparisons, due to the small size of the examined populations, the significance of any difference between the median values of the physiological/metabolic parameters as well as elemental concentrations were tested using the nonparametric Mann–Whitney U test.

To demonstrate the relationship between the examined physiological/metabolic parameters, Pearson’s or Spearman’s correlation was applied, depending on the data distribution (normal or not, respectively). Data distribution for each parameter was evaluated using the Shapiro-Wilk test. To examine the intragroup relationships between the determined chemical elements, Spearman’s correlation was used.

Statistical analysis was performed using Statistica 13.1 software (ver. 13.3, Tibco Software Inc.,Statsoft, Krakow, Polska). In all the statistical tests, results with a *p* value of less than 0.05 (5% level) were considered as being statistically significant.

### 4.6. Discriminant Analysis Based on of Tissue Elemental Concentration

Total reflection X-ray fluorescence analysis provides information of the multi-element content simultaneously. Therefore all the data were kept together to further highlight the differences between the obese and lean groups of animals using a multivariate statistical technique. To determine which elements contribute most to the discrimination between the groups of animals, an MDA was carried out. Due to the abnormal data distribution for selected elemental concentrations, all variables were transformed to log base 10 prior to the further steps of the MDA. The main criterion for the determination of the elements contributing significantly to the distinguishing of the animal groups was the minimization of Wilks’ lambda. The discriminant function for distinguishing between the lean and the obese rats was calculated by the forward stepwise procedure. For description of the significance of the primary variables (elemental concentrations), partial Wilks’ lambda (PWL) was used.

## 5. Conclusions

In the face of the increasing prevalence of obesity, any identification of early markers of adult body adiposity is useful for clinicians to prevent severe and irreversible health consequences and for help in further research to investigate the origin of obesity. Proper element metabolism plays a pivotal role in living organisms and excess or deficiency of biometals may change the function of organs and systems. Therefore, new tools and techniques such as Total Reflection X-ray Spectrometry are applied to detect these micronutrients in tissues and explain their role in the pathogenesis of diseases. Based on the results of our study, we robustly proposed Rb and confirmed FGF-19 and FGF-21 as new markers of excessive body adiposity and its metabolic alternations. However, new strategies must be developed to address this clinical issue and investigate the possible gender-related differences.

## Figures and Tables

**Table 1:** Partial Wilks’ Lambda (PWL) values for the determined elements for adipose tissue (A), muscle (M), kidney (K), liver (L), heart (H) and heart apex (HA).

Element	PWL_A_	PWL_M_	PWL_K_	PWL_L_	PWL_H_	PWL_HA_
K	- ^1^	0.68	-	-	**0.11**	0.78
Cr	0.76 ^2^	-	-	0.70	-	**0.31**
Fe	-	-	-	-	0.65	-
Cu	-	-	-	-	-	-
Zn	-	0.53	**0.54**	**0.36**	-	-
Se	-	-	0.78	-	-	0.51
Br	-	**0.49**	-	-	-	-
Rb	**0.064** ^3^	**0.013**	**0.037**	**0.087**	**0.023**	**0.33**
Sr	-	0.86	**0.47**	data	data	data

^1^ element out of model. ^2^ statistically insignificant value. ^3^ statistically significant values are given in bold.

## Data Availability

The data that support the findings of this study will be made available from the corresponding author after reasonable request up to 5 years after publication. We may require the participation in the authorship after the use of the shared data.

## Study Limitations

Only male subjects were used in our study, which was dictated by our experience that the male Wistar rats gain weight faster on HCD and are therefore a more reliable model of obesity. In addition, the vast majority of the literature cited in our study described the obtained results in males. This made it easier for us to compare and discuss the results obtained in our preliminary study. However, further research is encouraged to investigate the possible sex-related differences in measured parameters. Our current study focused on measuring selected biochemical, hormonal and elemental parameters to investigate if there is any relationship between obesity-related metabolic changes and elemental composition of specific organs and tissues. Having confirmed the validity of our hypothesis in our preliminary study, we could next focus on the correlation of possible molecular changes with possible early morphological alternations in organs using other techniques, e.g., histopathology, sub-inflammation detection in organ homogenates, etc. It should be noted, however, that macroscopically, apart from the features of liver steatosis (white spots infiltrating the liver tissue) and increased body adiposity, we did not observe any organ changes in obese subjects at this stage of obesity. Probably, additional information would expand our knowledge in this area, but this is a task for another research project.
